# Mechanism of Deep-Sea Fish α-Actin Pressure Tolerance Investigated by Molecular Dynamics Simulations

**DOI:** 10.1371/journal.pone.0085852

**Published:** 2014-01-20

**Authors:** Nobuhiko Wakai, Kazuhiro Takemura, Takami Morita, Akio Kitao

**Affiliations:** 1 Department of Computational Biology, Graduate School of Frontier Sciences, The University of Tokyo, Tokyo, Japan; 2 Institute of Molecular and Cellular Biosciences, The University of Tokyo, Tokyo, Japan; 3 Research Center for Fisheries Oceanography and Marine Ecosystem, National Research Institute of Fisheries Sciences, Fisheries Research Agency, Kanagawa, Japan; Jacobs University Bremen, Germany

## Abstract

The pressure tolerance of monomeric α-actin proteins from the deep-sea fish *Coryphaenoides armatus* and *C. yaquinae* was compared to that of non-deep-sea fish *C. acrolepis*, carp, and rabbit/human/chicken actins using molecular dynamics simulations at 0.1 and 60 MPa. The amino acid sequences of actins are highly conserved across a variety of species. The actins from *C. armatus* and *C. yaquinae* have the specific substitutions Q137K/V54A and Q137K/L67P, respectively, relative to *C. acrolepis*, and are pressure tolerant to depths of at least 6000 m. At high pressure, we observed significant changes in the salt bridge patterns in deep-sea fish actins, and these changes are expected to stabilize ATP binding and subdomain arrangement. Salt bridges between ATP and K137, formed in deep-sea fish actins, are expected to stabilize ATP binding even at high pressure. At high pressure, deep-sea fish actins also formed a greater total number of salt bridges than non-deep-sea fish actins owing to the formation of inter-helix/strand and inter-subdomain salt bridges. Free energy analysis suggests that deep-sea fish actins are stabilized to a greater degree by the conformational energy decrease associated with pressure effect.

## Introduction

**Figure 1:**
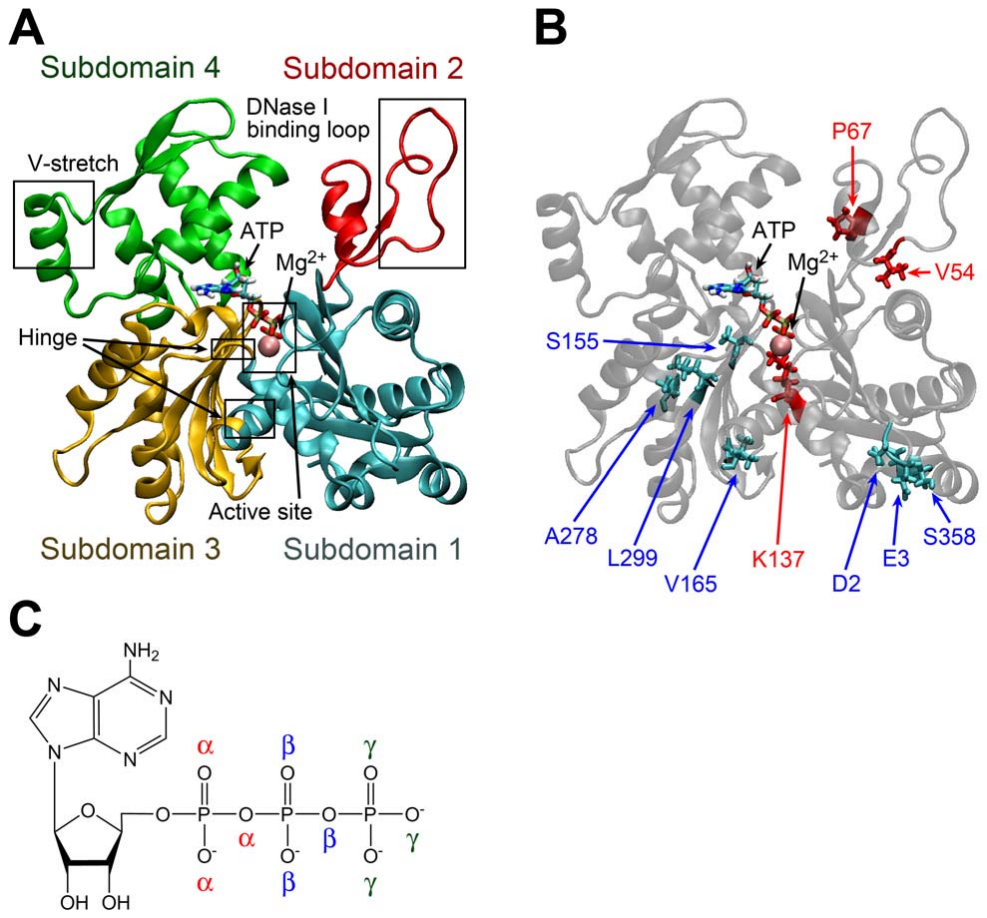
Structure of monomeric actin. (A) Subdomain arrangement. Subdomains 1, 2, 3 and 4 are shown in cyan, red, yellow and green, respectively. The pink sphere represents Mg^2+^ at the active site. (B) Positions of substituted residues in *C. yaquinae* actin as compared to rabbit/chicken actin. The residues shown in red and cyan in the licorice model represent the specific substitutions in deep-sea fish actins and those of terrestrial animals and shallow-water fish species, respectively. (C) Chemical formula of ATP. Oxygen atoms in the phosphate tail of ATP are distinguished by α, β, and γ.

Actin is a protein responsible for numerous cellular functions. For example, actin serves as a component of muscle fibers in muscle cells, supports cell structure, and is involved in cellular motility as a component of microfilaments even in non-muscle cells. The amino acid sequences of actin isoforms are highly conserved across a variety of species. The main component of muscle fiber is α-actin, which is one of the most abundant proteins. α-actin forms stable filaments that bind to myosin filaments in sarcomeres. Actin filaments are also polymerized and depolymerized as motor proteins, especially in filopodia and lobopodia [1]. The first X-ray crystal structure of monomeric globular-actin (G-actin) was solved in 1990 [2], and since then over 70 G-actin structures have been registered in the Protein Data Bank (PDB). Structure models of filamentous actin (F-actin) were formerly deduced from the structures of G-actin; however, a few filament structures were recently determined using electron cryomicroscopy [3], [4], [5], [6]. A divalent cation (typically Mg^2+^ or Ca^2+^) and a nucleotide (ATP or ADP) bind to the center of G-actin (Figure 1A). The divalent cation is coordinated with oxygen atoms of the phosphate groups at the tail of the nucleotide and with water molecules. The α-actin monomer consists of two major domains separated by the nucleotide binding site. The domain composed of subdomains 1 and 2 is arranged on the surface of actin filament, and another domain consisting of subdomains 3 and 4 forms the core of the filament [2]. Subdomain 2 contains a very flexible loop, which binds to DNase I [2] and a neighboring F-actin protomer. Other parts of the actin molecule also interact with many actin-binding proteins, such as profilin and cofilin [7], [8], which affect the actin conformation and accelerate and decelerate the rate of polymerization, respectively. Most of the actin-binding proteins are associated with nucleation and the formation or stabilization of F-actin.

Deep-sea fish can be found at depths down to ∼6000 m, where the pressure reaches ∼60 MPa. *Coryphaenoides*, a marine fish known as rattail or grenadier, inhabits a wide depth range, making it a good species for studying pressure tolerance. The *Coryphaenoides* species *C. acrolepis*, *C. armatus*, and *C. yaquinae* inhabit depths of around 180–2000, 2700–5000, and 4000–6400 m, respectively. The amino acid sequences of α-actins from these three species are known to be highly conserved, and actins of terrestrial animals or shallow-water fish species also have similar sequences [9], [10]. The rates of actin polymerization and ATP and Ca^2+^ dissociation in non-deep-sea fish are significantly affected by pressures but these rates in deep-sea fish are unaffected by pressures at least up to 60 MPa, even though the actin sequences differ by only a few residues from those of other species (Table 1). Deep-sea fish actins have a lysine as residue 137 (K137) near the active site, where other species have a glutamine (Q137). Residue Q137 is predicted to affect the hydrolysis reaction and is believed to be one of the key residues in the polymerization process based on evidence suggesting that it plays an important role in controlling water molecules that behave as nucleophiles and attack ATP [11]. The attack on ATP by water molecules can significantly impact the rate of polymerization [12]. In addition to the Q137K substitution, deep-sea fish actins have either L67P or V54A substitutions, both of which are distant from the active site and are located on the protein surface bound to neighboring F-actin protomers (Figure 1B), suggesting that they affect the pressure tolerance of F-actin polymerization. Therefore, Q137K is expected to play an essential role in the pressure tolerance of deep-sea fish α-actin because the position is near the active site of hydrolysis.

**Table 1:** Sequence features of the various actins examined in this study [10].

		Residue									
Species and type	Label	2	3	54	67	137	155	165	278	299	358
Rabbit/Chicken	Rab	E	D	V	L	Q	S	I	T	M	T
*C. acrolepis* actin 1^a^	Ac1W, Ac1Q^c^	D	E	V	L	Q	A	V	A	L	S
*C. acrolepis* actin 2a^a^	Ac2	D	E	V	L	Q	S	V	A	L	S
*C. armatus* actin 2b^b^	Arm	D	E	**A**	L	**K**	S	V	A	L	S
*C. yaquinae* actin 2b^b^	Yaq	D	E	V	**P**	**K**	S	V	A	L	S
Carp	–	D	D	V	L	Q	A	V	A	L	T

a Non-deep-sea fish actin.

b Deep-sea fish actin [10].

c See main text for the differences. Unlisted amino acid residues are identical.

At high pressure, protein denaturation, conformational changes, and loss of enzymatic activity are observed [10], [13], [14], [15]. The ligand dissociation rates of hydrolases and dehydrogenases were shown to increase at high pressure [10], [16], [17]. Fluctuation of hen egg white lysozyme in picosecond time range was also affected by pressure and temperature [18]. The effect of pressure on actin was first reported in 1966 [14]. Pressure-induced denaturation of rabbit G-actins begins to occur at 250 MPa and is complete at 400 MPa [14]. Pressure prevents G-actin from assembling due to denaturation or conformational changes [10]. High pressure has been shown to induce significant changes in actins purified from terrestrial animals or shallow-water fish species, as evidenced by a decrease in DNase I inhibition, decreased volume change upon polymerization, an increase in the critical concentration, and increases in ligand dissociation rates [10].

Molecular dynamics (MD) simulation is a powerful tool for investigating the effects of pressure on proteins. Several studies have used MD simulation to examine denaturation, conformational changes, water penetration, and volume changes in proteins under a wide range of pressures [19], [20], [21], [22]. In the case of ubiquitin, water penetration is induced at ∼300 MPa, and at higher pressures denaturation is observed [19]. Collapse of the secondary structure and an increase in the radius of gyration at high pressure were studied using the water-insertion method [22]. Changes in protein structure induced by high pressure can also be examined by calculating the solvent-accessible surface area (SASA) and volume through both experiments and simulations. NMR analyses and volume calculations based on atomic coordinates showed that high pressure compresses protein volume by only 1–3% [22]. Since most globular proteins form highly packed structures in the native state, a volume change of this magnitude is relatively small. Most high-pressure simulations involve pressures well over 100 MPa, which is sufficient to induce denaturation of many proteins. However, relatively few studies have addressed the effects of pressures below 100 MPa on protein structure.

In this work, the effects of pressure up to 60 MPa on the structure and dynamics of G-actin from two deep-sea fish (*C. armatus* and *C. yaquinae* actin 2b), two actins from non-deep-sea fish (*C. acrolepis* actin 1 and actin 2a), and rabbit/chicken actins were investigated using MD simulations at 0.1 and 60 MPa. Free energy analysis shows that deep-sea fish actins at high pressure are stabilized by a decrease in the conformational energy of actin without large change in the solute entropy of actin. We also report that only two amino acid differences are sufficient to induce significant changes in the pattern of salt bridging, which is suggested to play a significant role in stabilization of ATP binding and subdomain arrangement at high pressure. Possible differences in ATP hydrolysis mechanisms will be also discussed.

## Methods

### Structure Modeling of Actins

To analyze the effect of amino acid substitutions *in vivo*, it is necessary to obtain actin mutants. However, it is well-recognized that most mutant actins produced by mutagenesis cannot be expressed [12]. Therefore, almost all experiments in the previous studies were carried out using actins purified from muscle fiber. Instead of determining actin mutant structures, we conducted molecular modeling of the various actins based on the rabbit one *in silico*. Because the amino acid sequence of actin is highly conserved, this modeling was relatively straightforward. The actin molecules studied in this work are listed in Table 1. First, the atomic coordinates of rabbit skeletal muscle α-actin, including crystal water molecules, ATP, and Ca^2+^ were adopted from a high-resolution crystal structure (PDB entry 1WUA, resolution 1.45 Å [23]). Because residues 1–4 of the N-terminus, residues 42–50 of the flexible DNase I binding loop, and residues 372–375 of the C-terminus were missing in this structure, lower resolution structures from PDB entries 1ATN (resolution 2.80 Å) [2] and 1ESV (resolution 2.00 Å) [24] were employed for residues 1–4, 39–53, and 372–375, respectively. Hydrogen-atom coordinates were added using the visual molecular dynamics (VMD) software package [25]. The modeled rabbit actin coordinates were also employed as the template to model other actin structures. Actin 2b of the deep-sea fish species *Coryphaenoides armatus* and *C. yaquinae* are referred to as Arm and Yaq, respectively. Actin 1 and 2a of the non-deep-sea fish species *C. acrolepis* are labelled as Ac1 and Ac2, respectively. *C. acrolepis* actins contain 60% Ac2 and 40% Ac1. In contrast, actins of *C. armatus* consist of 20% Ac2 and 80% Arm, and those of *C. yaquinae* consist of 19% Ac2 and 81% Yaq. Of note, the sequence of rabbit actin (labelled as Rab) is identical to those of human and chicken actins.

Except for the Q137K substitution, the other fish actin substitutions relative to the rabbit/chicken actin sequence are located on the protein surface distant from the active site and distant from each other, making the modeling of these side chains straightforward. Because Q137K is located in the active site and is predicted to be important for pressure tolerance, we were careful in our modeling approach. All 81 rotamers were considered as possible side-chain structures of K137. After energy minimization, we compared the structures and chose the structure for which the Ca^2+^–N^ζ^ distance was the longest. The Ca^2+^ in the enzymatic pocket was replaced with Mg^2+^ to reproduce physiological conditions [26]. The N-terminus was modified with acetyl-aspartate, and 3-methylhistidine (3-MeH) was adopted for residue 73. In all actins except that of the ameba *Naegleria gruberi*, histidine 73 is known to be post-translationally modified to 3-MeH [27]. Of note, 3-MeH is protonated histidine with the methyl group located at position 3 in the imidazole ring. Because the N^δ1^ atom in 3-MeH can form a hydrogen bond with the carbonyl group of G158 (which forms a hydrogen bond with ATP), the hydrogen bond network of 3-MeH is associated with the ATP hydrolysis [27]. All five G-actin models examined (i.e., Yaq, Arm, Ac1, Ac2, and Rab) were solvated in a periodic boundary box with 50 mM KCl and a solvation water layer of at least 10 Å.

After a 3000-step energy minimization using the conjugate gradient method, MD simulations were first performed by keeping non-modeled protein atoms, K137, ATP, and Mg^2+^ fixed at the initial position for 1 ns and then restraining the same atoms with harmonic positional restraints for 1 ns. The force constraints were started from 1 kcal/mol/Å^2^ and gradually decreased by 0.1 kcal/mol/Å^2^ every 0.1 ns. After the restrained MD simulation, we began 32 MD simulations for both the Ac1 and Rab systems with distinct initial velocities and carried out the simulations independently at 0.1 MPa for 0.1 ns without restraints. We obtained two distinct Mg^2+^ coordination patterns in Ac1∶1) coordination by four water molecules and two γ-oxygen atoms of ATP in 26 cases (Ac1W), and 2) coordination by three water molecules, two γ-oxygen atoms of ATP, and a Q137 side-chain oxygen atom in six cases (Ac1Q). We examined cation coordination patterns for 73 globular α-actin structures in the PDB and found that 42 X-ray crystal structures had coordination patterns similar to that of Ac1W, whereas Ac1Q-type coordination was formed only in six relatively low-resolution structures with the following PDB ID resolutions: 1ATN [2], 2.80 Å; 1IJJ [28], 2.85 Å; 1LCU [28], 3.50 Å; 1H1V [29], 3.00 Å; 1RFQ [30], 3.00 Å; 1Y64 [31], 3.05 Å. The initial side-chain dihedral angles of residue 137, which had dihedral angles χ_1_ to χ_3_ from the C^α^ to the C^δ^ of the end of the side chain in Ac1W, were χ_1_ = −61.7°, χ_2_ = −176.5°, and χ_3_ = 177.3°. The dihedral angles for Ac1Q were χ_1_ = −58.1°, χ_2_ = 166.9°, and χ_3_ = −168.8°. Therefore, the initial difference between Ac1W and Ac1Q was primarily the χ_2_ and χ_3_ dihedral angles of residue 137. For each pattern, one representative structure was selected and investigated with longer MD simulations (Table 1). In all 32 Rab simulations, Q137 was not coordinated to Mg^2+^.

### Molecular Dynamics Simulations

A total of six different models (i.e., Ac1W, Ac1Q, Ac2, Rab, Arm, and Yaq) were simulated using MD at pressures of 0.1 and 60 MPa. The high-pressure MD simulation was conducted after gradually raising the pressure from 0.1 to 0.2 MPa in 0.03 ns and then to 60 MPa in 0.2-MPa increments per 0.03 ns. MD simulations were performed with the CHARMM22 force field [32], [33], [34] and SPC/E water model [35] using the NAMD software package [36]. The parameter files were modified to accommodate 3-MeH [37]. SPC/E model was employed because its translational diffusion constant and the rotational correlation time are the closest to the experiment values among TIP3P, TIP4P, SPC, and SPC/E [38]. The 3-MeH parameters and topology were generated in the CHARMM22 files using a doubly protonated histidine and an N-methylamide C-terminus patch [39]. Electrostatic potentials were calculated using the particle mesh Ewald procedure [40], and van der Waals interactions were computed using a 12-Å cutoff and a smooth switching function. The simulations were conducted with periodic boundary conditions in an isobaric-isothermal ensemble, with the exception of the initial 2-ns simulations, in which a canonical ensemble (constant *NVT*) was employed. Constant temperature was maintained using Langevin dynamics for non-hydrogen atoms with a damping coefficient of 5 ps^−1^, whereas constant pressure was maintained using a Langevin piston [41] with an oscillation period of 100 fs and a decay time of 50 fs. The bond between each hydrogen and the atom to which it is bonded in the solute is constrained using the SHAKE algorithm [42], and the internal geometry of water molecules was kept rigid using the SETTLE algorithm [43]. To reproduce experimental conditions, MD simulations were carried out at 277 K and either 0.1 or 60 MPa. This temperature was selected to reproduce the condition in the experiment [10]. This temperature is typically used to reproduce the deep-sea environment in the field. Deep-sea temperatures are 1–4°C [44]. A possible little difference between the simulation and real temperatures (<3°C) is expected to be within the range of fluctuation and its effects should be very small. The MD time step of 2 fs was used for the simulations, which were performed for 100 ns (50-ns equilibration and 50-ns sampling). The coordinates and energy data were stored every 0.5 and 0.1 ps, respectively.

### Analysis of Physical Properties

To examine the effects of high pressure on G-actin, we calculated the excluded volume (*V*_ex_), SASA, and the isothermal compressibility (*κ_T_*). Both *V*_ex_ and SASA were calculated using the CAVE software package [45]. SASA is defined by the track of the probe center as the probe rolls around the whole surface of the protein, and the space inside the track of the probe is defined as *V*_ex_. The probe radius was 1.4 Å, and the van der Waals radius was used for each protein atom. These van der Waals radii were 2.0 Å for the sp^3^ carbon, sp^3^ nitrogen, and sulfur with a hydrogen, 1.7 Å for sp^2^ carbon without hydrogen and sp^2^ nitrogen with a hydrogen, 1.85 Å for sp^2^ carbon with hydrogen(s) and sulfur without hydrogen, 1.8 Å for sp^2^ nitrogen with hydrogens and 1.4 Å for oxygen [46].

Protein compressibility is a property that is associated with the structure of a protein. The packing density of a protein is non-uniform due to the presence of small cavities. Because compressibility is significantly affected by internal cavities and hydration, it is an effective measure of protein structure property. Compressibility is also related to toughness and fluctuation. The adiabatic compressibility of various proteins has been measured experimentally using the velocity of sound in solution [47], and from these measurements the isothermal compressibility can be estimated. We calculated the isothermal compressibility, *κ_T_*, which is defined as follows.
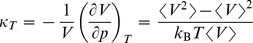
(1)where *V*, *p*, *k*_B_, and *T* represent the system volume, pressure, the Boltzmann constant, and the absolute temperature, respectively. The angle bracket denotes the average over last 50 ns of the simulation.

To understand stability of actin at high pressure, free energy shift Δ*G* caused by pressure change from 0.1 to 60 MPa was estimated using the following thermodynamic cycle,
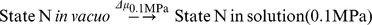


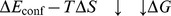


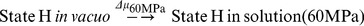
(2)where states N and H represent stable states of actin at normal (0.1 MPa) and high (60 MPa) pressure, respectively. In this conceptual cycle, we consider that solvation steps do not alter actin structures in states N and H. Δ*μ*_0.1 MPa_ and Δ*μ*_60 MPa_ indicate solvation free energies, i.e., transfer free energies from vacuum to solution at 0.1 and 60 MPa, respectively. Δ*E*_conf_ and Δ*S* are change of conformational energy and solute entropy from states N to H. From the cycle shown by (2), Δ*G* can be calculated as,



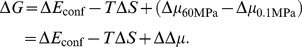
(3)Δ*E*_conf_ was calculated as change of the average conformational energy of solute (actin) from states N to H using the NAMD software package [36]. As shown in the cycle (2), the solvation steps are defined not to alter actin structures. Therefore, Δ*E*_conf_ *in vacuo* is calculated using snapshots of MD simulations in solution. Δ*μ* was estimated by the method proposed in the reference [48] and is divided into two contributions,
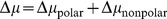
(4)where Δ*µ*_polar_ and Δ*µ*_nonpolar_ are the polar and nonpolar solvation free energy, respectively. Δ*µ*_polar_ was estimated by calculating the Poisson dielectric continuum model using DelPhi software package [49] with water dielectric constants estimated using the Harris and Alder *g*-factor [50] at the simulated temperature and pressure. Δ*µ*_nonpolar_ can be approximately decomposed into the contributions from three components [51],
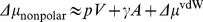
(5)where γ, A, and ΔµvdW are the surface tension, surface area, and free energy of van der Waals attraction. From the values given in [52], γ at 277 K was set to 0.1091 kcal/mol/Å2. We ignored ΔµvdW term because its contribution to yield improvements less than 0.1% [48]. Solute entropy, S, is calculated by the total sum of entropy in the translational (Strans), rotational (Srot), and internal motion (Sint) [53].
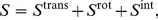
(6)which are defined by,



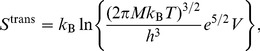
(7)

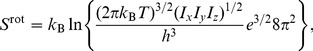
(8)

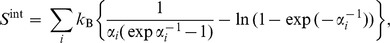


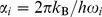
(9)where *h*, *M*, *V*, and *ω_i_* are Plank constant, mass of protein, volume in L/mol, and angular frequency of normal mode, respectively. *I_x_*, *I_y_*, and *I_z_* denote protein principal moments of inertia. *S* ^int^ is deduced from the covariance matrix of coordinates, similar to so-called configurational entropy [54]. The *ω_i_* is calculated as the effective frequency of principal mode [55] obtained by the principal component analysis of simulations using mass-weighted all-atom coordinates. Equation (9) of *S* ^int^ includes the kinetic term whereas the configurational entropy has only configurational integral of potential term. Assuming that states N and H are invariant in the process of solvation in the thermodynamics cycle, we calculated solute entropy from the MD trajectories in solution. Last 50-ns simulation was divided into five 10-ns simulations. We independently calculated the entropy using these 10-ns simulations and then obtained the average entropies of actins.

All molecular images shown in figures were generated using VMD software package [25].

## Results and Discussion

### Comparison of Structure and Fluctuation

First, the average structures resulting from last 50-ns trajectories of 12 MD simulations were compared based on the mutual root mean-square deviation (RMSD) of the backbone heavy atoms. The maximum RMSD in all 66 combinations was 2.5 Å between Arm and Rab at 0.1 MPa, whereas the minimum RMSD was 1.5 Å between Arm and Ac1Q at 0.1 MPa. The average RMSD was 2.0±0.3 Å. For deep-sea fish actins, the RMSDs between high- and low-pressure structures were 1.5 and 2.3 Å for Yaq and Arm, respectively. For non-deep-sea fish actins, the RMSDs between high- and low-pressure structures were in the range 2.0–2.2 Å. There were no significant differences between the different actins with respect to the magnitude of the average conformational change induced by high pressure.

The “propeller angle” defined by the relative rotation between subdomains was shown to change upon polymerization to F-actin [4]. We calculated the propeller angle as the torsion angle defined by the centers of four subdomains (Table S1). Variations of the propeller angle among the species and shift of the average propeller angle caused by high pressure were not largely different from the standard deviations.

**Figure 2:**
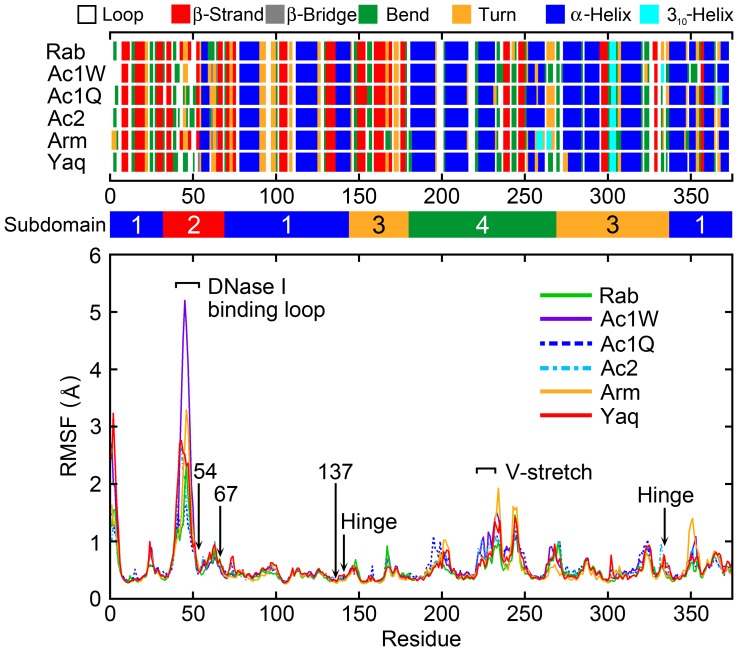
The root mean-square fluctuation (RMSF) per residue at 60 MPa. The RMSF was calculated by best-fitting the backbone heavy atoms of each snapshot to the average structure. Secondary structure and subdomain assignments are also shown.

The root mean-square fluctuation (RMSF) per residues from the average structure using actin backbone heavy atoms at 60 MPa is shown in Figure 2. The most flexible region is the DNase I binding loop (residues 42–55) of subdomain 2. This region had a notably high RMSF compared with all other regions. The V-stretch region (residues 227–237) corresponded to the second highest peak except for the N-terminal region peak. The V-stretch consists of one α-helix (residues 222–233) of subdomain 4 exposed in G-actin, which also make no contact with adjacent protomers in F-actin. RMSFs of the substituted residues in deep-sea fish actins (residues V54, P67, and K137) were very small (RMSF <1 Å). Figure 2 also shows the secondary structure profiles of the actins at 60 MPa. Simulations of the DNase I binding loop returned a variety of secondary structures because this region is very flexible. The position of the α-helices and β-strands in the deep-sea fish actins corresponded to those of the non-deep-sea fish actins except for some end regions of the α-helix and β-strand. Although high pressure affects actin function, the secondary structures were well-maintained even under high pressure.

### Analysis of *V*_ex_, SASA, and *κ_T_*

As representative quantities to examine the effects of pressure, *V*_ex_, SASA, and *κ_T_* were calculated and are shown in Table 2. No notable differences were observed between deep-sea and non-deep-sea fish actins with respect to these parameters. Although *V*_ex_ tended to decrease slightly at higher pressure, the differences were comparable to the standard deviations, as were the differences in the values for SASA. Denaturation associated with high pressure generally induces a decrease in *V*_ex_ and an increase in SASA due to protein unfolding; however, the pressures examined in this work were much lower than that necessary for denaturation of actin, which begins to occur at 250 MPa [14]. A positive globular protein adiabatic compressibility suggests a more compact structure at high pressure. Ultrasonic measurements indicate that the adiabatic compressibilities of filamentous proteins including F-actin and myosin are negative, which confirm that this property is related to the hydration of protomer surfaces [56]. In this work, the *κ_T_* of the various actins examined was in the range 0.13–0.15 GPa^−1^; no clear systematic differences were observed. The reported *κ_T_* values as determined from sound velocity measurements for 25 globular proteins whose molecular weights ranged from 12,400–232,000 were in the range 0.0192–0.150 GPa^−1^ [57]. The results also indicated that larger proteins are more compressible. The molecular weight of actin is ∼41,800. The reported *κ_T_* values of comparable seven proteins with a molecular weight between 30,000 and 70,000 is in the range 0.0932–0.150 GPa^−1^ except for peroxidase whose *κ_T_* is exceptionally small (0.0670 GPa^−1^). Therefore, the *κ_T_* of actin is comparable to that of proteins of similar size. Of note, the *κ_T_* for pure water determined from sound velocity measurements at different pressures is 49.175 Mbar^−1^ (0.49175 GPa^−1^) at 278 K and 0.1 MPa and 41.912 Mbar^−1^ (0.41912 GPa^−1^) at 278 K and 60 MPa [58].

**Table 2:** Effect of high pressure on excluded volume (*V*_ex_), solvent accessible surface area (SASA) and isothermal compressibility (*κ_T_*).

	*V*_ex_			SASA			*κ_T_*		
Label	0.1 MPa	60 MPa	Δ	0.1 MPa	60 MPa	Δ	0.1 MPa	60 MPa	Δ
Rab	7.39±0.02	7.37±0.02	−0.02	1.82±0.02	1.84±0.02	0.02	0.14±0.02	0.13±0.02	−0.01
Ac1W	7.40±0.02	7.41±0.03	0.01	1.84±0.02	1.87±0.02	0.03	0.14±0.02	0.15±0.04	0.01
Ac1Q	7.40±0.02	7.39±0.02	−0.01	1.84±0.02	1.86±0.03	0.02	0.14±0.02	0.14±0.01	−0.01
Ac2	7.41±0.03	7.36±0.02	−0.05	1.84±0.02	1.83±0.02	−0.01	0.15±0.04	0.14±0.02	−0.01
**Arm**	**7.39±0.02**	**7.36±0.03**	**−0.03**	**1.83±0.02**	**1.84±0.02**	**0.01**	**0.13±0.02**	**0.15±0.04**	**0.02**
**Yaq**	**7.42±0.02**	**7.40±0.02**	**−0.02**	**1.85±0.02**	**1.87±0.02**	**0.02**	**0.13±0.02**	**0.15±0.03**	**0.02**

Units: *V*_ex_ (10^4^ Å^3^), SASA (10^4^ Å^2^), *κ_T_*, (GPa^−1^). Δ = *X*_60 MPa_ – *X*_0.1 MPa_ where *X* = *V*_ex_, SASA, or *κ_T_*. The value after “±” indicates standard deviation.

### Free Energy Analysis

Energy shifts caused by high pressure (Table 3) were examined by the method described in Methods section. Details of each energy term are shown in Table S2, S3, S4. Free energy differences between 60 and 0.1 MPa (Δ*G*) were all positive, which indicate that actin at 60 MPa are less stable compared to 0.1 MPa. Δ*G* values of Arm and Yaq were the lowest and second lowest, respectively, and were significantly lower than the others. This is consistent to the fact that Arm and Yaq are stable at high pressure. Δ*E*_conf_ were significantly negative for Arm and Yaq, which were the main cause of the stabilization of Arm and Yaq. Contributions of electrostatic interactions in Δ*E*_conf_ were −158±69 (Arm) and −167±99 kcal/mol (Yaq), which were dominant term in Δ*E*_conf_. The results indicate that deep-sea fish actins at high pressure are stabilized by the conformational energy decrease.

**Table 3:** Energy differences between 60 and 0.1

Label	Δ*E*_conf_	ΔΔ*μ*	*T*Δ*S*	Δ*G*	ΔΔ*G*
Rab	−59±53	520±32	18±18	444±57	−135
Ac1W	11±103	532±14	16±26	527±122	−51
Ac1Q	16±85	575±43	13±15	579±65	0
Ac2	−52±127	512±29	19±24	441±112	−138
**Arm**	**−147±67**	**510±22**	**29±20**	**334±69**	**−244**
**Yaq**	**−153±92**	**535±25**	**30±11**	**352±77**	**−226**

Unit: kcal/mol. Δ*X* = *X*_60 MPa_ – *X*_0.1 MPa_ where *X* = *E*_conf_, Δ*μ*, *TS*, or *G*. Δ*G* = Δ*E*_conf_+ΔΔ*μ* − *T*Δ*S*. ΔΔ*G* is the difference from Δ*G* of Ac1Q.

### Hydrogen Bond and Salt Bridge Analyses

The results of the free energy analysis suggest that intra-solute interaction in deep-sea fish actins is a key to understand high pressure tolerance. To examine this, we conducted hydrogen bond and salt bridge analyses. The number of hydrogen bonds in actin monomers and between actin monomers and water was shown in Table S5. Within actin, variations in the hydrogen-bond numbers among the species and shift of the average hydrogen-bond numbers caused by high pressure were not largely different from the standard deviations. The number of hydrogen bonds between actin and water molecules showed more variations among the species. This quantity can be related to solvation free energy, rather than conformational energy. The number of hydrogen bonds increased at high pressure in all the cases. There was no clear correlation between the number of hydrogen bonds and pressure tolerance.

Table 4 shows the number of salt bridges between ATP and surrounding residues. A salt bridge was considered to be formed if the distance between oxygen and nitrogen atoms of charged groups was less than or equal to 3.2 Å [25]. It should be noted that multiple salt bridges can be formed in a pair of residues with this definition. Deep-sea fish actins have K137 at the active site, whereas non-deep-sea fish actins have Q137. The Q137K substitution in deep-sea fish actin changes the charge at this position from neutral to positive. Deep-sea fish actins formed a salt bridge between the γ-oxygen atoms of ATP (Figure 1C) and the side chain of K137 at 0.1 and 60 MPa. Deep-sea fish actins formed more salt bridges between ATP and the surrounding residues than non-deep-sea fish actins. Ac2 had the least number of salt bridges at 60 MPa. Although deep-sea fish actins include some proportion of Ac2 (20% in *C. armatus* and 19% in *C. yaquinae*), Ac2 is found predominantly in non-deep-sea fish actins (60% in *C. acrolepis*). The ligand dissociation rate constants of non-deep-sea fish actins were shown to increase notably at high pressure, whereas those of deep-sea fish actins are less affected [10]. The salt bridge between ATP and K137 is expected to stabilize ATP binding at high pressure, thus enhancing the protein’s pressure tolerance. Residue K137 in deep-sea fish actins is located near the hinge region (residues 141–142 or 336–337) of the propeller motion. All of the subdomains also make contact with the active site. Furthermore, transformation of G- to F-actin is associated with the hydrolysis of ATP. Therefore, a conformational change in the ATP γ-phosphate bound to K137 is expected to play a large role in affecting the change in actin structure occurring upon filament formation. The Q137K substitution might trigger a propagation of the changes in protein conformation and salt bridge pattern.

**Table 4:** Number of salt bridges formed between ATP and surrounding residues.

Label	K18-O_α_	K18-O_β_	K137-O_γ_	Total
**0.1 MPa**				
Rab	1.0±0.0	1.5±0.6	–	2.5±0.6
Ac1W	0.8±0.4	1.4±0.6	–	2.2±0.7
Ac1Q	0.7±0.6	1.1±0.6	–	1.8±0.8
Ac2	1.0±0.1	1.4±0.6	–	2.4±0.6
**Arm**	**1.0±0.2**	**0.6±0.6**	**1.1±0.4**	**2.7±0.8**
**Yaq**	**1.0±0.1**	**1.0±0.3**	**1.7±0.5**	**3.7±0.5**
**60 MPa**				
Rab	1.0±0.0	1.1±0.7	–	2.1±0.7
Ac1W	1.0±0.0	1.4±0.5	–	2.4±0.5
Ac1Q	1.0±0.0	1.3±0.5	–	2.3±0.5
Ac2	0.3±0.5	1.0±0.1	–	1.3±0.5
**Arm**	**1.0±0.0**	**0.9±0.3**	**1.4±0.5**	**3.3±0.6**
**Yaq**	**1.0±0.2**	**0.8±0.6**	**1.0±0.3**	**2.8±0.7**

K18-O_α_, K18-O_β_, and K137-O_γ_ represent salt bridges between K18 and α-oxygen, between K18 and β-oxygen, and between K137 and γ-oxygen of ATP, respectively. The value after “±” indicates standard deviation.

**Figure 3:**
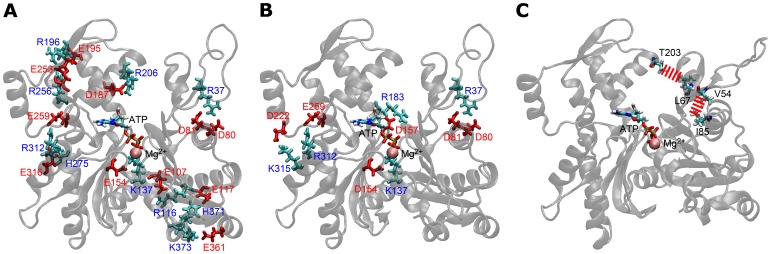
Salt bridge and hydrophobic interactions in actin. The salt bridges (A) between secondary structures and (B) between subdomains in Yaq at 60 MPa. The residues that form salt bridges with a formation rate of more than 0.5 are shown in Yaq at 60 MPa. Red and blue represent acidic and basic amino acids, respectively. (C) Hydrophobic interactions involving specific substituted residues in Ac1 actin. Red broken lines indicate the hydrophobic interaction. Residues 54 and 67 are different in the actins of deep-sea fish and other species.

Table 5 shows the number of salt bridges formed between secondary structures and subdomains. Let us first focus on differences in the number of salt bridges among different species. Deep-sea fish actins tended to form more salt bridges than the actins of other species. With the exception of Yaq and Arm, the rank order with respect to the total number of salt bridges formed corresponded to the experimentally determined rank order with respect to pressure tolerance (i.e., Yaq was the most pressure tolerant, followed by Arm, Rab, Ac2, and Ac1). The differences of the total number of salt bridges between Yaq and Arm at 0.1 and 60 MPa were within the range of standard deviations. These data suggest that the number of salt bridges formed is closely related to the degree of pressure tolerance. Deep-sea fish actins also formed more inter-helix/strand salt bridges than non-deep-sea fish actins. The number of intra-helix/strand salt bridges formed by the various actins was comparable, except for Rab, which tended to form more salt bridges within the helix and strand at high pressure (Table 5). Figure 3A shows the positions of residues involved in the formation inter-helix/strand salt bridges. These residues are expected to stabilize the arrangements between secondary structures at multiple sites in deep-sea fish actins. The number of inter- and intra-subdomain salt bridges is also shown in Table 5. It is clear that deep-sea fish actins form more inter-subdomain salt bridges than do actins from other species, and this might play a role in stabilizing the subdomain arrangement. It should be noted that no inter-subdomain salt bridges were found between subdomains 2 and 4 (Figure 3B). Therefore these inter-subdomain salt bridges do not interfere with propeller angle rotation. Changes in the number of salt bridges induced by high pressure were also seen in Table 5. The average total number of salt bridges did not largely change in Rab, Arm and Yaq (From −0.2 to +0.6 changes whereas the standard deviations are 2.6–3.1) that are relatively high pressure tolerant, but larger increase in the salt bridge number (From +3.7 to +6.0 increases whereas the standard deviations are 2.3–3.8) was observed in Ac1W, Ac1Q and Ac2 that are less tolerant to pressure. The results indicate that only small change in terms of salt bridges was caused by high pressure in Rab, Arm and Yaq, showing the robustness of these actins.

**Table 5:** Number of salt bridges formed between secondary structures and subdomains.

	Secondary structure							ATP			Subdomain					
Label	Inter helix/strand^a^			Helix/strand and loop^b^	Loop and loop^c^	Intra helix/strand^d^		ATP and residues^e^			Intersubdomain^f^			Intrasubdomain^g^		Total^h^
**0.1 MPa**																
Rab	16.4±1.5		9.9±2.1		0.0±0.0	11.3±1.7	2.5±0.6			10.2±1.4			27.4±2.6		40.2±3.1	
Ac1W	14.7±1.5		6.9±1.5		0.2±0.4	6.8±1.3	2.2±0.7			8.0±0.9			20.5±2.0		30.7±2.3	
Ac1Q	11.9±2.1		8.4±1.5		0.4±0.5	6.7±1.4	1.8±0.8			7.4±1.3			20.0±2.4		29.2±2.8	
Ac2	12.8±1.8		8.5±2.1		0.7±0.9	8.0±1.7	2.4±0.6			7.0±1.4			23.0±3.2		32.4±3.8	
**Arm**	**20.5±1.6**		**9.2±1.4**		**1.0±1.2**	**8.9±1.4**	**2.7±0.8**			**10.7±1.3**			**28.9±2.4**		**42.4±2.6**	
**Yaq**	**19.1±1.8**		**9.0±2.6**		**0.1±0.3**	**9.5±1.7**	**3.7±0.5**			**11.1±1.4**			**26.6±3.5**		**41.4±4.4**	
**60 MPa**																
Rab	15.9±1.5	11.2±1.9			0.0±0.1	11.6±2.2	2.1±0.7		9.2±1.5			29.4±2.8			40.8±2.9	
Ac1W	15.7±1.4	9.9±2.2			0.0±0.0	6.4±1.2	2.4±0.5		8.2±1.4			23.8±2.7			34.4±3.2	
Ac1Q	14.1±1.4	8.9±1.6			0.0±0.2	7.9±1.4	2.3±0.5		7.9±1.0			23.1±2.6			34.2±2.4	
Ac2	14.7±1.8	11.1±1.5			2.3±0.7	9.0±1.6	1.3±0.5		9.1±1.3			28.0±2.2			38.4±2.6	
**Arm**	**19.4±1.4**	**11.1±1.6**			**0.0±0.0**	**8.3±2.1**	**3.3±0.6**		**11.5±1.3**			**27.4±2.5**			**42.2±2.8**	
**Yaq**	**18.8±1.6**	**11.8±1.8**			**0.2±0.4**	**8.4±1.7**	**2.8±0.7**		**10.5±1.2**			**28.7±2.6**			**41.9±2.6**	

Salt bridges ^a^between distinct helices or strands, ^b^between a helix/strand and a loop, ^c^between distinct loops, ^d^within helix or strand. ^e^Salt bridges between ATP and a residue. ^f^Inter and ^g^intra subdomain salt bridge. ^h^The sum of “Secondary structure”+“ATP” or “Subdomains”+“ATP”. The value after “±” indicates standard deviation.

### Possible ATP Hydrolysis Mechanism

**Figure 4:**
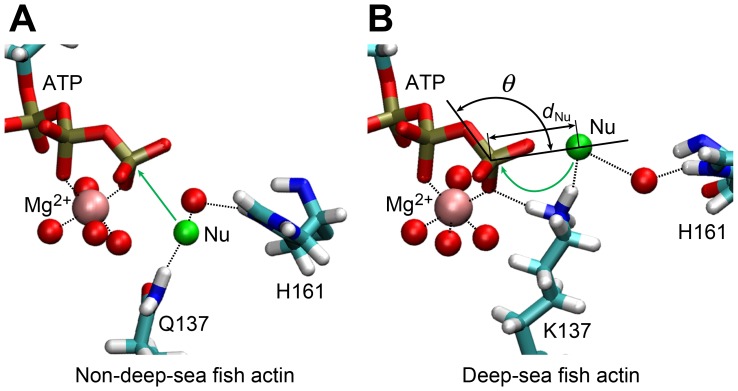
Arrangement of the water molecule expected to initiate nucleophilic attack on the γ-phosphate of ATP. The arrangement of non-deep-sea fish actins (A) and deep-sea fish actins (B). Green spheres show water molecules expected to be nucleophilic water for ATP hydrolysis. Red spheres indicate the water molecules coordinated to Mg^2+^ and those bridging the expected nucleophilic water and H161 with hydrogen bonds. Black dotted lines show typical hydrogen bonds formed during the MD simulation. Angle *θ* and distance *d*_Nu_ are defined by O^β^-P^γ^-O^w^ and P^γ^-O^w^, respectively, where O^w^ represents the oxygen of the expected nucleophilic water (see Figure 1C for the definition of the other atoms).

It was reported that the Q137A mutant actin polymerized four times faster than wild-type actin, but cleavage of the ATP γ-phosphate group occurred at only one-fourth of the rate of wild-type actin, indicating the residue 137 has a significant effect on these processes [12]. We observed that the difference in the amino acid residue at residue 137 between deep-sea fish and non-deep-sea fish actins also alters the orientation of the side chain. Actin hydrolyzes ATP mainly in F-actin elongation process and the enzyme activity is very weak in G-actin. The nucleophilic water attacks the γ-phosphate of ATP during the hydrolysis. The inactive nucleophilic water is probably held by the residue 137 and the water molecule bound to H161 [59] (Figure 4). Residue 137 is located in subdomain 1, whereas H161 is located in subdomain 3. It was suggested that H161 is moved by the conformational changes that occur when the adjacent actin protomer makes contact and ATP hydrolysis subsequently begins [4]. In all of the MD simulations, we confirmed that ATP binds Mg^2+^ at the active site tightly, with a coordination number of 6.0 for all of the actins examined.

In yeast G-actin, the energy barrier of hydrolysis neglecting entropic contribution was 28.8 kcal/mol estimated using the minimum energy path of quantum chemical calculations [59]. A QM/MM model of ATP hydrolysis without protein was carried out including the entropic contribution and the energy barrier was 33.4 kcal/mol [60]. Since ATP hydrolysis in enzyme active sites is not investigated using QM/MM models with the entropic contribution, the detailed energy of the hydrolysis is unclear yet. We considered the effect of K137 binding to the γ-phosphate group. In general, a divalent cation assists the process of ATP hydrolysis. If K137 is bound to the O^γ^ in deep-sea fish actin during the hydrolysis as observed in the MD, more positive charge is coordinated to the O^γ^ and the energy barrier of the hydrolysis could be lowered in the intermediate state. In this case, the rate of hydrolysis can be accelerated in deep-sea fish actins; however, the rate of hydrolysis in actins of deep-sea fish and non-deep-sea fish are comparable at low pressure [10]. Therefore, Q137K substitution probably has also disadvantage for the hydrolysis.

**Figure 5:**
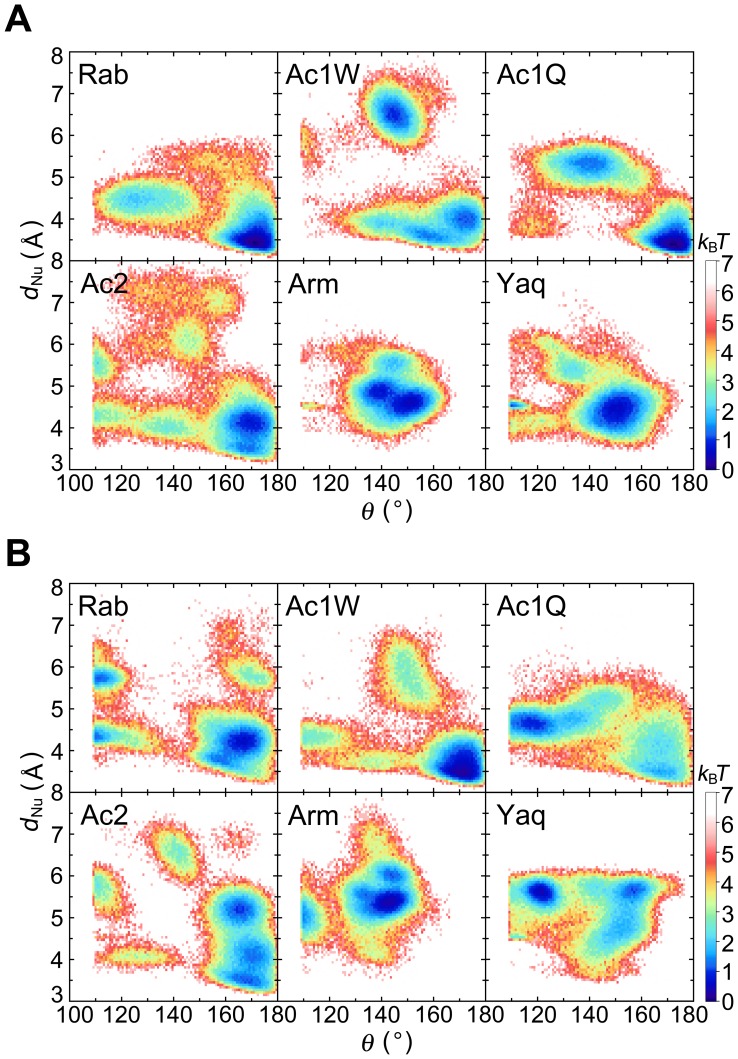
Spatial distribution of expected nucleophilic water. Distribution of expected nucleophilic water as a function of angle *θ* and distance *d*_Nu_, as defined in the legend for Figure 4, converted as free energy scale at (A) 0.1 and (B) 60 MPa. A water molecule having the minimum *d*_Nu_ value and a *θ* greater than 109.3° was assigned as the expected nucleophilic water in each simulation snapshot. The free energy was shown as the relative value against the minimum free energy in *k*_B_ *T*.

We focused on the positions of water molecules in the active site. Figure 5 indicates the distribution of the expected nucleophilic water in the hydrolysis (see Figure 5 legend for the definition of expected nucleophilic water) shown by free energy scale as the function of the angle *θ* and distance *d*_Nu_, which are defined by O^β^-P^γ^-O^w^ and P^γ^-O^w^, respectively, where O^w^ is the oxygen atom of the expected nucleophilic water molecule. The free energy minimum in deep-sea fish actins was around 150° at 0.1 MPa, whereas in non-deep-sea fish actins the free energy minimum was around 170° (Figure 5A). Since the nucleophilic water linearly attacks the γ-phosphate of ATP (*θ* = ∼180°), non-deep-sea fish actins at 0.1 MPa maintained one water molecule at the favorable position for nucleophilic attack. This in-line arrangement was also observed in a previous study of MD simulations [61]. At 60 MPa, the *θ* angle had a free energy minimum at around 160–170° in non-deep-sea fish actins and around 140–150° in deep-sea fish actins (Figure 5B and Table S6). In deep-sea fish actins, residue K137 is directly bound to the γ-oxygen atoms of ATP and expected nucleophilic water (Figure 4). The P^γ^-O^w^ distance (*d*_Nu_) was 4.6 Å or less in each of the non-deep-sea fish actins at 60 MPa and 5.3–5.5 Å in the deep-sea fish actins (Table S6). Thus, the expected nucleophilic water in deep-sea fish actins is slightly shifted both in *θ* and *d*_Nu_ from the best in-line position. The stabilization of the γ-phosphate group in the intermediate state with more positive charge is suggested to be compensated with the effect of the less favorable position of the expected nucleophilic water.

### Effect of V54A and L67P

Deep-sea fish actins have either a V54A (Arm) or L67P (Yaq) substitution at the surface of subdomain 2. Because the DNase I binding loop of subdomain 2 binds to neighboring actin protomers in F-actin, these substitutions are also expected to contribute to pressure tolerance. Although these two residues are relatively close to protomer-protomer interface in F-actin, they do not directly interact with other protomers. The V54A and L67P substitutions do not significantly alter the polarity, but they do alter the hydrophobicity of the protein to a certain extent. The Kyte-Doolittle hydropathy indexes for valine, alanine, leucine, proline, isoleucine, and threonine are 4.2, 1.8, 3.8, −1.6, 4.5, and −0.7, respectively; that of isoleucine is the highest among the standard amino acids [62]. Both V54 and L67 are highly hydrophobic residues in non-deep-sea fish actins, whereas deep-sea fish actins have less hydrophobic residues at these positions (i.e., A at position 54 and P at position 67). Residues V54 and L67 in non-deep-sea fish actins are directed toward neighboring subdomains and can interact with I85 and T203, respectively (Figure 3C).

Residue I85 is located in subdomain 1 near the boundary with subdomain 2. A strong hydrophobic interaction between the side chains of residues V54 and I85 was formed in all actins examined except Arm. The minimum distance between the side-chain carbon atoms (*d*_54–85_) at 0.1 and 60 MPa was 3.9 and 3.8–3.9 Å, respectively, whereas the *d*_54–85_ for A54 and I85 at 0.1 and 60 MPa in Arm was 4.4 and 4.3 Å, respectively (Table S7). Because alanine is smaller and less hydrophobic than valine, the *d*_54–85_ in Arm was slightly longer than that in other actins. In spite of slightly weaker hydrophobic interaction, there was no change in this distance at 0.1 and 60 MPa in any of the actins examined. The Arm DNase I binding loop had a tendency to form additional β-strand structure at residues 43–44 and 48–49 compared with actins other than Rab (Figure 2). The tendency might have some effect on the protomer-protomer interaction in F-actin.

The L67P substitution in Yaq is located at the surface of subdomain 2 near the DNase I binding loop and nucleotide binding cleft between subdomains 2 and 4 (Figure 1B). T203 is the nearest residue to residue 67 in subdomain 4. The minimum distance between the side-chain carbon atoms of residues 67 and 203 (*d*_67–203_) in Arm and Yaq at 60 MPa was 6.2±1.0 and 6.8±0.7 Å, respectively (Table S7). In non-deep-sea fish actins, *d*_67–203_ had more variations among different actins at 60 MPa (4.3 [Rab]-7.1 Å [Ac1Q]).

In summary, the effect of V54A and L67P substitutions on pressure tolerance was not clear in the G-actin simulation. This effect should be examined with F-actin simulation.

### Comparison between Two Actin 1a Models

In this work, we considered two actin 1a structures, Ac1W and Ac1Q. In the crystal structures, the high-affinity cation binding site consists of a divalent cation, chelate water molecules, and some side-chain atoms. Of the 48 PDB structures having chelating groups at the α-actin active site (PDB files as of June 8, 2013), only six have Q137 as the chelating group. In this sense, the coordination state represented by Ac1W predominates that represented by Ac1Q among the PDB structures. A comparison of the MD simulation results showed that the two coordination states are closely related, both structurally and dynamically. Indeed, the physical properties and salt bridge patterns were almost the same, except for the salt bridge between K18 and the β-oxygen atom of ATP. This salt bridge would slightly attract the phosphate tail to the region included in Q137, also affecting the expected nucleophilic water position in Ac1. The minimum energy coordination of the nucleophilic water in Ac1W at 60 MPa was at *θ* = ∼170° and *d*_Nu_ = ∼3.0 Å, whereas at *θ* = ∼120° and *d*_Nu_ = ∼4.3 Å in Ac1Q. This difference indicates that Ac1W maintains the nucleophilic water at a more favorable in-line position. Since the Mg^2+^ in Ac1Q is maintained at an unfavorable position for attacking the γ-phosphate of ATP, direct coordination of Q137 to Mg^2+^ is probably disadvantageous for ATP hydrolysis. This is also consistent with the cases of Ac2 and Rab, which do not involve binding between Q137 and Mg^2+^ at the active site.

We also analyzed the dihedral angle of residue 137, which had dihedral angles χ_1_ to χ_3_. At 0.1 MPa, the χ_3_ of Ac1Q bound to Mg^2+^ was significantly different than that of Ac1W. The peaks of the dihedral angles in Ac1Q differed at 0.1 and 60 MPa, whereas we did not observe a significant difference in the dihedral angles at 0.1 and 60 MPa in Ac1W (Figure S1). Additional peaks were found both in χ_1_ (−44° and ±180°) and χ_3_ (88°) at 60 MPa in Ac1Q. Presumably, at 60 MPa the conformation of residue 137 in Ac1Q is unstable, which is consistent with the fact that the crystal structures included in the bond between Q137 and Mg^2+^ are minor. Δ*G* of Ac1Q was larger than that of Ac1W and was the largest among the actins studied (Table 3). Therefore, Ac1W can be considered as a more plausible model for Ac1.

## Conclusions

Deep-sea fish actins from *C. armatus* (Arm) and *C. yaquinae* (Yaq) have specific substitutions (Q137K and V54A [Arm] or I67P [Yaq]) not found in actins of terrestrial animals or species of shallow-water fish. Although the pressure of the deep-sea habitat is below the actin denaturation pressure, pressure has significant effects on polymerization and the dissociation rates of ATP and Ca^2+^ in non-deep-sea fish actins, whereas the actins of deep-sea fish are tolerant of pressures up to at least 60 MPa [10], [15]. In this work, we investigated the effect of the amino acid substitutions on pressure tolerance using MD simulations. We found that high pressure causes only small changes in the excluded volume, SASA, isothermal compressibility, solvation energy, and solute entropy, of both deep-sea and non-deep-sea fish actins, but conformational energy of Arm and Yaq actins significantly lowered at high pressure. Therefore, we conclude that deep-sea fish actins at high pressure are stabilized in the conformational energy decrease. Salt bridge pattern of Arm and Yaq showed notable differences compared to the others studied in this work. The salt bridges between ATP and K137, which were only formed in deep-sea fish actins, are expected to stabilize ATP binding even under high pressure. Deep-sea fish actins also formed a greater total number of salt bridges than non-deep-sea fish actins, owing to the formation of inter-helix/strand and inter-subdomain salt bridges at high pressure. Therefore, we conclude that two amino acid differences are sufficient to significantly stabilize ATP binding and subunit arrangement through the salt bridges.

The residue differences may also affect polymerization of G-actin into F-actin. Because the change in the propeller angle is related to subdomain rearrangement upon polymerization, residue K137, which is located near the hinge of the propeller motion in deep-sea fish actin, should also affect polymerization. The effect of the amino acid substitution on F-actin polymerization should be also investigated by MD simulations in the future.

## Supporting Information

Figure S1 **Probability distribution of dihedral angles of residue 137 in Ac1W and Ac1Q.** The red, blue, and green lines denote the χ_1_, χ_2_, and χ_3_ dihedral angles of residue 137 in Ac1, respectively.
(TIF)

Table S1 **Propeller angle defined by the actin subdomains.**
(DOC)

Table S2 **Effect of high pressure on actin conformational energy.**
(DOC)

Table S3 **Effect of high pressure on solute entropy.**
(DOC)

Table S4 **Effect of high pressure on actin solvation energy.**
(DOC)

Table S5 **The number of hydrogen bonds in actin and between actin and water.**
(DOC)

Table S6 **Coordination of the expected nucleophilic water to ATP.**
(DOC)

Table S7 **Minimum inter-residue distances at V54A and L67P.**
(DOC)
