# Supplementary figures and images for: Mechanism of Deep-Sea Fish α-Actin Pressure Tolerance Investigated by Molecular Dynamics Simulations
Source: PLoS One. 2014 Jan 20;9(1):e85852. doi: 10.1371/journal.pone.0085852 (PMC3896411; doi:10.1371/journal.pone.0085852)

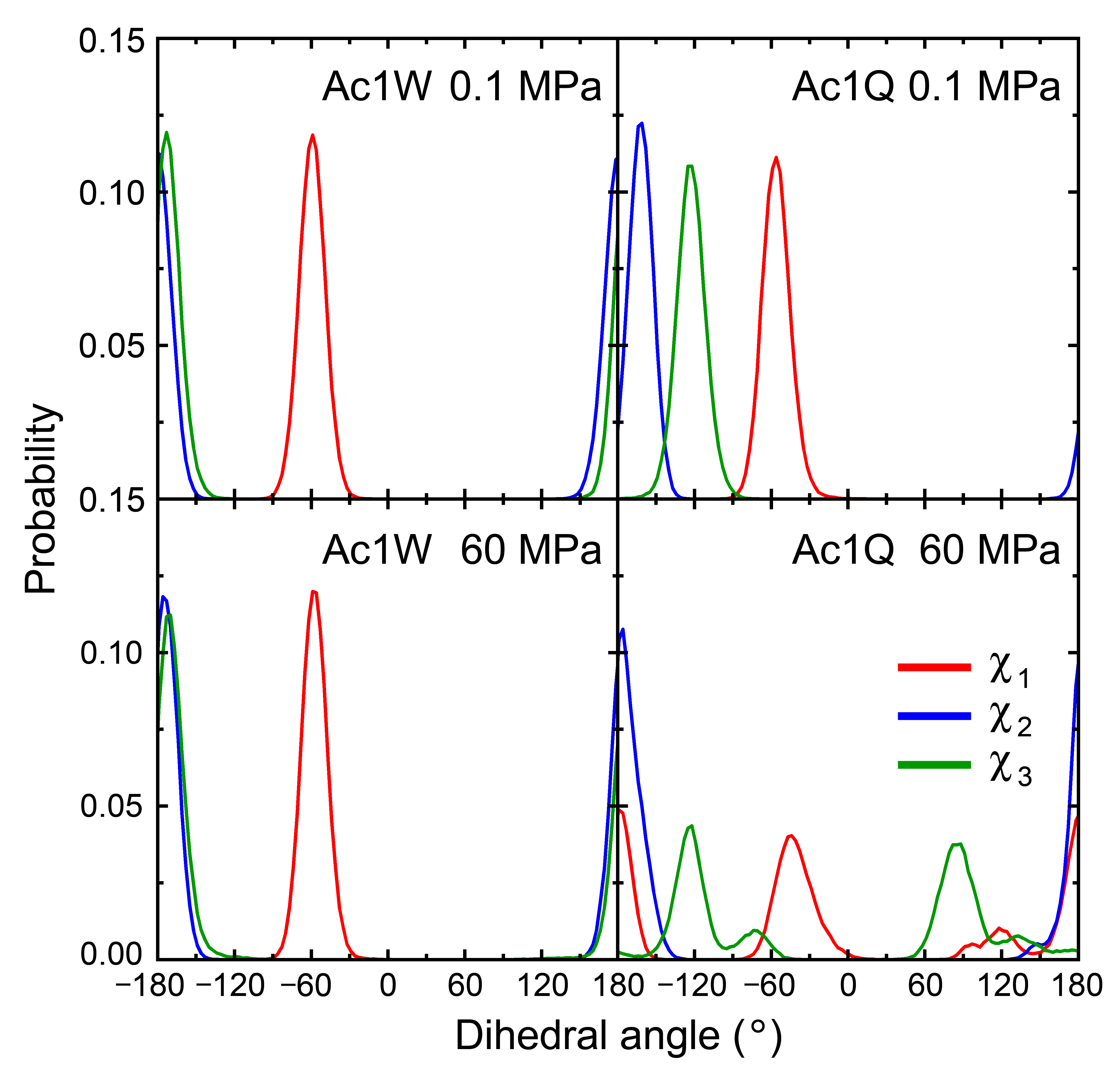

Supplement: Figure S1 — Probability distribution of dihedral angles of residue 137 in Ac1W and Ac1Q. The red, blue, and green lines denote the χ1, χ2, and χ3 dihedral angles of residue 137 in Ac1, respectively. (TIF) [file pone.0085852.s001.tif]
