# Supplementary material for: Mechanism of Deep-Sea Fish α-Actin Pressure Tolerance Investigated by Molecular Dynamics Simulations
Source: PLoS One. 2014 Jan 20;9(1):e85852. doi: 10.1371/journal.pone.0085852 (PMC3896411; doi:10.1371/journal.pone.0085852)
Supplement: Table S1 — Propeller angle defined by the actin subdomains. (DOC) [file pone.0085852.s002.doc]

| **Table S1.** Propeller angle defined by the actin subdomains. | | | |
| --- | --- | --- | --- |
|  | | | |
|  | **Propeller angle (˚)** | | |
| **Label** | **0.1 MPa** | **60 MPa** | **Δ** |
| Rab | 16.9 ± 1.9 | 17.8 ± 3.9 | 0.8 ± 4.4 |
| Ac1W | 20.2 ± 1.9 | 22.7 ± 2.6 | 2.5 ± 3.2 |
| Ac1Q | 18.0 ± 1.7 | 21.1 ± 3.3 | 3.1 ± 3.7 |
| Ac2 | 20.7 ± 2.0 | 21.1 ± 2.7 | 0.4 ± 3.4 |
| **Arm** | **17.6 ± 1.8** | **21.3 ± 2.0** | **3.7 ± 2.7** |
| **Yaq** | **17.5 ± 2.2** | **14.8 ± 2.5** | **−2.6 ± 3.4** |
| Δ = (Propeller angle)_60MPa_ – (Propeller angle)_0.1MPa_. The value after “±” indicates standard deviation. | | | |
