# Supplementary material for: Mechanism of Deep-Sea Fish α-Actin Pressure Tolerance Investigated by Molecular Dynamics Simulations
Source: PLoS One. 2014 Jan 20;9(1):e85852. doi: 10.1371/journal.pone.0085852 (PMC3896411; doi:10.1371/journal.pone.0085852)
Supplement: Table S2 — Effect of high pressure on actin conformational energy. (DOC) [file pone.0085852.s003.doc]

| **Table S2.** Effect of high pressure on solute energy. | | | |
| --- | --- | --- | --- |
|  | | | |
|  | ***E*_conf_ (kcal/mol)** | | |
| **Label** | **0.1 MPa** | **60 MPa** | **Δ** |
| Rab | −3735 ± 41 | −3794 ± 33 | −59 ± 53 |
| Ac1W | −3632 ± 68 | −3621 ± 77 | 11 ± 103 |
| Ac1Q | −3705 ± 52 | −3688 ± 68 | 16 ± 85 |
| Ac2 | −3612 ± 123 | −3664 ± 30 | −52 ± 127 |
| **Arm** | **−3578 ± 44** | **−3725 ± 50** | **−147 ± 67** |
| **Yaq** | **−3543 ± 82** | **−3696 ± 41** | **−153 ± 92** |
| Δ = (*E*_conf_)_60MPa_ – (*E*_conf_)_0.1MPa_. The value after “±” indicates standard deviation. | | | |
