# Supplementary material for: Mechanism of Deep-Sea Fish α-Actin Pressure Tolerance Investigated by Molecular Dynamics Simulations
Source: PLoS One. 2014 Jan 20;9(1):e85852. doi: 10.1371/journal.pone.0085852 (PMC3896411; doi:10.1371/journal.pone.0085852)
Supplement: Table S3 — Effect of high pressure on solute entropy. (DOC) [file pone.0085852.s004.doc]

| **Table S3.** Effect of high pressure on solute entropy. | | | | | | | | | | |
| --- | --- | --- | --- | --- | --- | --- | --- | --- | --- | --- |
|  | | | | | | | | | | |
|  | **0.1 MPa** | | | |  | **60 MPa** | | | |  |
| **Label** | ***TS*^trans^** | ***TS*^rot^** | ***TS*^int^** | ***TS*** |  | ***TS*^trans^** | ***TS*^rot^** | ***TS*^int^** | ***TS*** | **Δ*TS*** |
| Rab | 126 ± 0 | 104 ± 0 | 4432 ± 11 | 4662 ± 11 |  | 126 ± 0 | 104 ± 0 | 4450 ± 15 | 4680 ± 15 | 18 ± 18 |
| Ac1W | 126 ± 0 | 104 ± 0 | 4413 ± 19 | 4643 ± 19 |  | 126 ± 0 | 104 ± 0 | 4429 ± 18 | 4659 ± 18 | 16 ± 26 |
| Ac1Q | 126 ± 0 | 104 ± 0 | 4400 ± 12 | 4630 ± 12 |  | 126 ± 0 | 104 ± 0 | 4413 ± 8 | 4642 ± 8 | 13 ± 15 |
| Ac2 | 126 ± 0 | 104 ± 0 | 4382 ± 8 | 4612 ± 8 |  | 126 ± 0 | 104 ± 0 | 4402 ± 23 | 4631 ± 23 | 19 ± 24 |
| **Arm** | **126 ± 0** | **104 ± 0** | **4405 ± 14** | **4635 ± 14** |  | **126 ± 0** | **104 ± 0** | **4434 ± 14** | **4664 ± 14** | **29 ± 20** |
| **Yaq** | **126 ± 0** | **104 ± 0** | **4393 ± 10** | **4623 ± 10** |  | **126 ± 0** | **104 ± 0** | **4423 ± 4** | **4653 ± 4** | **30 ± 11** |
| Unit: kcal/mol. Δ*TS*= *TS*_60MPa_ – *TS*_0.1MPa_. The value after “±” indicates standard deviation. | | | | | | | | | | |
