# Supplementary material for: Mechanism of Deep-Sea Fish α-Actin Pressure Tolerance Investigated by Molecular Dynamics Simulations
Source: PLoS One. 2014 Jan 20;9(1):e85852. doi: 10.1371/journal.pone.0085852 (PMC3896411; doi:10.1371/journal.pone.0085852)
Supplement: Table S4 — Effect of high pressure on actin solvation energy. (DOC) [file pone.0085852.s005.doc]

| **Table S4.** Effect of high pressure on actin solvation energy. | | | | | | | |
| --- | --- | --- | --- | --- | --- | --- | --- |
|  | | | | | | | |
|  | **0.1 MPa** | | |  | **60 MPa** | | |
| **Label** | **Δ*μ*_polar_** | **Δ*μ*_nonpolar_** | **Δ*μ*** |  | **Δ*μ*_polar_** | **Δ*μ*_nonpolar_** | **Δ*μ*** |
| Rab | −4638 ± 10 | 1952 ± 10 | −2686 ± 13 |  | −4771 ± 14 | 2606 ± 17 | −2165 ± 29 |
| Ac1W | −4663 ± 15 | 1971 ± 10 | −2691 ± 10 |  | −4801 ± 16 | 2642 ± 22 | −2159 ± 10 |
| Ac1Q | −4666 ± 15 | 1971 ± 11 | −2696 ± 11 |  | −4757 ± 23 | 2636 ± 23 | −2121 ± 43 |
| Ac2 | −4681 ± 35 | 1978 ± 10 | −2703 ± 27 |  | −4789 ± 6 | 2598 ± 14 | −2191 ± 10 |
| **Arm** | **−4657 ± 8** | **1962 ± 11** | **−2695 ± 18** |  | **−4793 ± 14** | **2608 ± 13** | **−2185 ± 13** |
| **Yaq** | **−4670 ± 24** | **1990 ± 15** | **−2680 ± 22** |  | **−4791 ± 7** | **2647 ± 14** | **−2144 ± 12** |
|  | | | | | | | |
| **Label** | **ΔΔ*μ*_polar_** | **ΔΔ*μ*_nonpolar_** | **ΔΔ*μ*** |  |  |  |  |
| Rab | −133 ± 17 | 653 ± 20 | 520 ± 32 |  |  |  |  |
| Ac1W | −139 ± 22 | 671 ± 25 | 532 ± 14 |  |  |  |  |
| Ac1Q | −91 ± 28 | 666 ± 25 | 575 ± 44 |  |  |  |  |
| Ac2 | −107 ± 35 | 619 ± 17 | 512 ± 29 |  |  |  |  |
| **Arm** | **−136 ± 16** | **646 ± 17** | **510 ± 22** |  |  |  |  |
| **Yaq** | **−121 ± 25** | **657 ± 20** | **535 ± 25** |  |  |  |  |
| Unit: kcal/mol. ΔΔ*X* = *X*_60MPa_ – *X*_0.1MPa_, *X* = Δ*μ*_polar_, Δ*μ*_nonpolar_, or Δ*μ*. The value after “±” indicates standard deviation. | | | | | | | |
