# Supplementary material for: Mechanism of Deep-Sea Fish α-Actin Pressure Tolerance Investigated by Molecular Dynamics Simulations
Source: PLoS One. 2014 Jan 20;9(1):e85852. doi: 10.1371/journal.pone.0085852 (PMC3896411; doi:10.1371/journal.pone.0085852)
Supplement: Table S5 — The number of hydrogen bonds in actin and between actin and water. (DOC) [file pone.0085852.s006.doc]

| **Table S5.** The number of hydrogen bonds in actin and between actin and water. | | | | | | | |
| --- | --- | --- | --- | --- | --- | --- | --- |
|  | | | | | | | |
|  | **The number of hydrogen bonds**  **within actin** | | |  | **The number of hydrogen bonds**  **between actin and water** | | |
| **Label** | **0.1 MPa** | **60 MPa** | **Δ** |  | **0.1 MPa** | **60 MPa** | **Δ** |
| Rab | 273 ± 6 | 272 ± 6 | −1 ± 8 |  | 874 ± 24 | 937 ± 16 | 63 ± 29 |
| Ac1W | 273 ± 6 | 265 ± 6 | −8 ± 8 |  | 927 ± 16 | 953 ± 21 | 26 ± 26 |
| Ac1Q | 273 ± 6 | 269 ± 6 | −4 ± 8 |  | 913 ± 23 | 939 ± 15 | 27 ± 27 |
| Ac2 | 271 ± 6 | 272 ± 6 | 2 ± 8 |  | 995 ± 99 | 1011 ± 95 | 16 ± 137 |
| **Arm** | **273 ± 7** | **274 ± 6** | **1 ± 9** |  | **922 ± 15** | **955 ± 22** | **33 ± 26** |
| **Yaq** | **268 ± 6** | **265 ± 6** | **−3 ± 8** |  | **926 ± 17** | **957 ± 15** | **31 ± 23** |
| Δ = (Hydrogen bond)_60MPa_ – (Hydrogen bond)_0.1MPa_. The value after “±” indicates standard deviation. | | | | | | | |
