# Supplementary material for: Mechanism of Deep-Sea Fish α-Actin Pressure Tolerance Investigated by Molecular Dynamics Simulations
Source: PLoS One. 2014 Jan 20;9(1):e85852. doi: 10.1371/journal.pone.0085852 (PMC3896411; doi:10.1371/journal.pone.0085852)
Supplement: Table S6 — Coordination of the expected nucleophilic water to ATP. (DOC) [file pone.0085852.s007.doc]

| **Table S6.** Coordination of the expected nucleophilic water to ATP. | | | | | | | | | | | |
| --- | --- | --- | --- | --- | --- | --- | --- | --- | --- | --- | --- |
|  | | | | | | | | | | | |
|  | ***θ*** | | |  | ***d*_Nu_** | | |  | ***d*_side_** | | |
| **Label** | **0.1 MPa** | **60 MPa** | **Δ** |  | **0.1 MPa** | **60 MPa** | **Δ** |  | **0.1 MPa** | **60 MPa** | **Δ** |
| Rab | 159 ± 19 | 154 ± 21 | −5 ± 28 |  | 4.0 ± 0.6 | 4.5 ± 0.7 | 0.5 ± 0.9 |  | 4.0 ± 0.6 | 3.9 ± 1.1 | −0.1 ± 1.3 |
| Ac1W | 154 ± 14 | 162 ± 16 | 8 ± 21 |  | 4.9 ± 1.3 | 4.1 ± 0.8 | −0.9 ± 1.5 |  | 3.3 ± 0.8 | 3.3 ± 0.7 | 0.0 ± 1.0 |
| Ac1Q | 158 ± 18 | 142 ± 21 | −16 ± 27 |  | 4.3 ± 0.9 | 4.5 ± 0.5 | 0.2 ± 1.0 |  | 3.4 ± 0.5 | 3.4 ± 0.6 | 0.0 ± 0.8 |
| Ac2 | 157 ± 19 | 161 ± 16 | 4 ± 24 |  | 4.5 ± 1.0 | 4.6 ± 0.9 | 0.1 ± 1.4 |  | 3.6 ± 1.2 | 3.3 ± 1.3 | −0.3 ± 1.8 |
| **Arm** | **145 ± 8** | **137 ± 11** | **−7 ± 13** |  | **4.8 ± 0.4** | **5.5 ± 0.5** | **0.7 ± 0.7** |  | **3.8 ± 0.6** | **4.7 ± 1.1** | **0.9 ± 1.2** |
| **Yaq** | **147 ± 12** | **139 ± 16** | **−7 ± 20** |  | **4.6 ± 0.5** | **5.3 ± 0.5** | **0.7 ± 0.7** |  | **3.4 ± 0.8** | **4.0 ± 0.6** | **0.6 ± 1.0** |
| Units: *θ* (˚), *d*_Nu_ and *d*_side_ (Å). The value after “±” indicates standard deviation. | | | | | | | | | | | |
