# Supplementary material for: Mechanism of Deep-Sea Fish α-Actin Pressure Tolerance Investigated by Molecular Dynamics Simulations
Source: PLoS One. 2014 Jan 20;9(1):e85852. doi: 10.1371/journal.pone.0085852 (PMC3896411; doi:10.1371/journal.pone.0085852)
Supplement: Table S7 — Minimum inter-residue distances at V54A and L67P. (DOC) [file pone.0085852.s008.doc]

| **Table S7.** Minimum inter-residue distances at V54A and L67P. | | | | | | | |
| --- | --- | --- | --- | --- | --- | --- | --- |
|  | | | | | | | |
|  | ***d*_54-85_** | | |  | ***d*_67-203_** | | |
| **Label** | **0.1 MPa** | **60 MPa** | **Δ** |  | **0.1 MPa** | **60 MPa** | **Δ** |
| Rab | 3.9 ± 0.2 | 3.9 ± 0.2 | 0.0 ± 0.3 |  | 4.5 ± 0.7 | 4.3 ± 0.9 | −0.2 ± 1.1 |
| Ac1W | 3.9 ± 0.3 | 3.9 ± 0.3 | 0.0 ± 0.4 |  | 7.0 ± 0.6 | 7.0 ± 0.9 | 0.0 ± 1.1 |
| Ac1Q | 3.9 ± 0.3 | 3.9 ± 0.3 | 0.0 ± 0.4 |  | 7.5 ± 0.6 | 7.1 ± 0.8 | −0.4 ± 1.0 |
| Ac2 | 3.9 ± 0.3 | 3.9 ± 0.3 | 0.0 ± 0.4 |  | 4.0 ± 0.4 | 5.0 ± 1.5 | 1.0 ± 1.6 |
| **Arm** | **4.4 ± 0.4** | **4.3 ± 0.3** | **−0.1 ± 0.5** |  | **8.1 ± 0.7** | **6.2 ± 1.0** | **−1.9 ± 1.2** |
| **Yaq** | **3.9 ± 0.3** | **3.8 ± 0.2** | **−0.1 ± 0.4** |  | **7.5 ± 0.4** | **6.8 ± 0.7** | **−0.7 ± 0.8** |
| Unit: Å. Δ = *X*_60MPa_ – *X*_0.1MPa_, *X* = *d*_54-85_ or *d*_67-203_. The value after “±” indicates standard deviation. | | | | | | | |
